# Emulsion and liposome-based adjuvanted R21 vaccine formulations mediate protection against malaria through distinct immune mechanisms

**DOI:** 10.1016/j.xcrm.2023.101245

**Published:** 2023-10-31

**Authors:** Sören Reinke, Eirini Pantazi, Gabrielle R. Chappell, Alexandra Sanchez-Martinez, Romain Guyon, Joannah R. Fergusson, Ahmed M. Salman, Anjum Aktar, Ekta Mukhopadhyay, Roland A. Ventura, Floriane Auderset, Patrice M. Dubois, Nicolas Collin, Adrian V.S. Hill, Jelena S. Bezbradica, Anita Milicic

**Affiliations:** 1 The Jenner Institute, Nuffield Department of Medicine, University of Oxford, Oxford OX3 7DQ, UK; 2 Kennedy Institute of Rheumatology Research, Nuffield Department of Orthopaedics, Rheumatology and Musculoskeletal Sciences, Medical Sciences Division, University of Oxford, Oxford OX3 7FY, UK; 3 Vaccine Formulation Institute, Rue du Champ-Blanchod 4, 1228 Plan-Les-Ouates, Switzerland

**Keywords:** adjuvants, vaccines, malaria, inflammasome, NLRP3, liposomes, emulsions, saponin, TLR4, QS-21

## Abstract

Adjuvanted protein vaccines offer high efficacy, yet most potent adjuvants remain proprietary. Several adjuvant compounds are being developed by the Vaccine Formulation Institute in Switzerland for global open access clinical use. In the context of the R21 malaria vaccine, in a mouse challenge model, we characterize the efficacy and mechanism of action of four Vaccine Formulation Institute adjuvants: two liposomal (LQ and LMQ) and two squalene emulsion-based adjuvants (SQ and SMQ), containing QS-21 saponin (Q) and optionally a synthetic TLR4 agonist (M). Two R21 vaccine formulations, R21/LMQ and R21/SQ, offer the highest protection (81%–100%), yet they trigger different innate sensing mechanisms in macrophages with LMQ, but not SQ, activating the NLRP3 inflammasome. The resulting *in vivo* adaptive responses have a different T_H_1/T_H_2 balance and engage divergent innate pathways while retaining high protective efficacy. We describe how modular changes in vaccine formulation allow for the dissection of the underlying immune pathways, enabling future mechanistically informed vaccine design.

## Highlights

• Two open access adjuvants offer equally strong protection against malaria in mice
• They induce T_H_1- or T_H_2-skewed response through divergent TLR4 and NLRP3 activation
• Modular adjuvant changes can balance cell lytic activity with protective efficacy
• Targeting distinct innate immune pathways can be used to polarize adaptive immunity

## Graphical abstract

**Figure:**
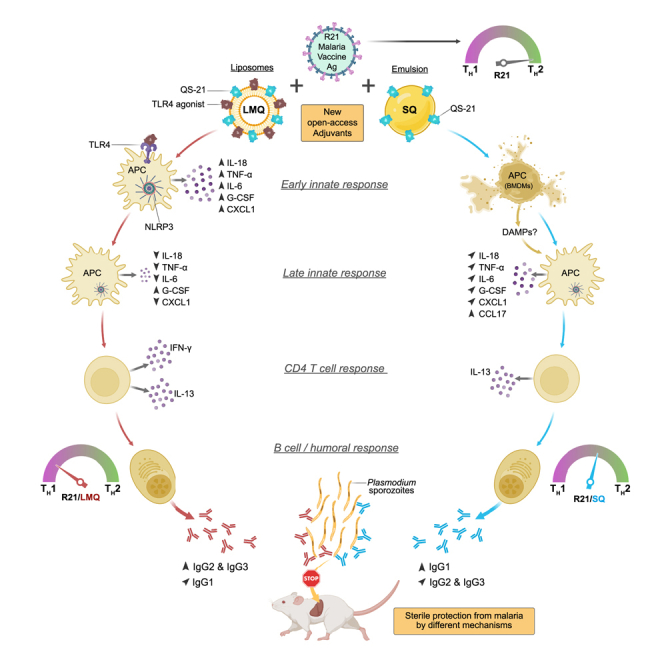

## Teaser

Reinke, Pantazi, et al. describe two safe open access adjuvants that, in combination with the R21 malaria vaccine, offer strong efficacy in mice. The liposome-based LMQ and squalene emulsion-based SQ differentially trigger the TLR4/NLRP3 inflammasome pathway, resulting in divergent Th1/Th2 responses, yet both ultimately lead to equally protective adaptive immunity.

## Introduction

The COVID-19 pandemic brought into sharp focus the need for potent vaccines that offer durable efficacy and can be deployed on equitable basis. Vaccine modalities such as mRNA and viral vectors have recently revealed their potential, demonstrating that a high degree of protection against infectious disease can be achieved through a range of distinct immunization approaches, likely engaging diverse immune pathways.^1^^,^^2^

Adjuvanted vaccines are a powerful platform, best illustrated by AS01 in Shingrix in older individuals and Mosquirix in African children.^3^^,^^4^ Adjuvanted protein vaccines have been shown to offer higher efficacy^5^ and more potent T follicular helper responses than viral vectors in malaria.^6^ By modulating the immune response to the vaccine antigen, adjuvants can enable vaccine dose sparing,^7^ induce broader immunity, and provide stronger protection in malnourished children and individuals with chronic viral infections,^8^^,^^9^ both of major concern in developing countries. However, most adjuvants are still largely proprietary, with only a few currently part of licensed human vaccines.^10^^,^^11^

To accelerate access to potent vaccine adjuvants, the Vaccine Formulation Institute (VFI) in Switzerland is developing openly available adjuvants for global use. Based on either liposomes or squalene oil-in-water emulsion, these adjuvants are supplemented with one or more immuno-stimulating compounds such as saponin or pattern recognition receptor agonists. This modularity, while providing wide-ranging flexibility for tailored vaccine design, also requires a detailed understanding of the adjuvant mechanism of action (MoA) to facilitate a plug-and-play approach.

We studied the MoA of four VFI adjuvants combined with our leading R21 malaria vaccine antigen (Ag), which, in combination with Matrix-M adjuvant, is currently in phase 3 trials, having demonstrated 77% protection in a phase 2 study in Africa,^12^^,^^13^ thereby meeting the WHO-specified 75% efficacy goal.^14^ In a mouse model of malaria, we tested R21 with two liposomal adjuvants (LQ, LMQ) and two squalene emulsion adjuvants (SQ, SMQ), all containing saponin QS-21, and LMQ and SMQ additionally supplemented with a synthetic MPL-like TLR4 agonist. Strong protection against developing malaria, matching that of R21/Matrix-M (R21/MM), was induced by R21 formulated in either LMQ or SQ, despite the two formulations triggering different innate and adaptive immune responses *in vivo*.

Our findings reveal a modular approach for mechanistically informed vaccine design and offer fresh insights into achieving vaccine protection through multiple immune pathways.

## Results

### LMQ and SQ promote strong protection against malaria through distinct humoral responses

**Figure 1:**
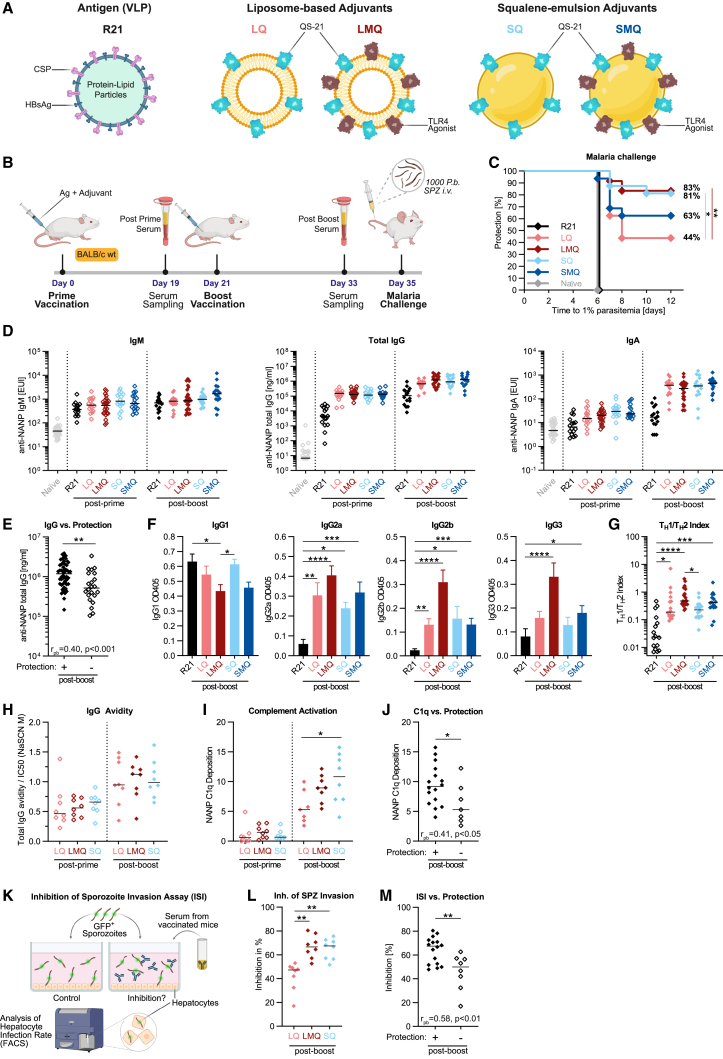
LMQ and SQ promote strong protection against malaria through distinct humoral responses (A) Schematic of R21 antigen and adjuvants LQ, LMQ, SQ, and SMQ. (B) Summary of the experimental protocol. Vaccine dose: 1 μg of R21; 25 μL of each adjuvant (containing 5 μg of QS-21 saponin with or without 2 μg of TLR4 agonist 3D6AP). (C) Survival post-malaria challenge: BALB/c mice were vaccinated and challenged as in (B). Parasitemia was assessed by daily blood smears with 1% parasitemia taken as irreversible malaria infection. ∗p < 0.05, ∗∗p < 0.01, log rank (Mantel-Cox) test. (C), (D), (F) and (G) show pooled data from three independent experiments; n = 16 R21, LQ, SQ, SMQ; n = 24 naive, LMQ. ∗p < 0.05, ∗∗p < 0.01. 01. Each symbol represents an individual mouse. (D) Serum anti-NANP IgG, IgM, and IgA isotype titers were assessed by ELISA. (E) Serum anti-NANP total IgG titers of mice protected (+, n = 50) vs. unprotected (−, n = 22) from malaria challenge across all groups. Data pooled from three independent experiments; median + replicates; ∗∗p < 0.01, Mann-Whitney test. Correlation analysis for (E), (J), and (M) was performed by point-biserial correlation; r_pb_ and p values are shown in the panels. (F) Anti-NANP IgG subclasses by proportional ELISA (Mean + SEM). (G) T_H_1/T_H_2 index of adjuvant-induced IgG subclasses calculated as ([IgG2a + IgG3]/2)/IgG1. Increased T_H_1/T_H_2 index indicates T_H_1-skewed immune response (median + replicates; ∗p < 0.05, ∗∗p < 0.01). (H) Total anti-NANP IgG avidity, measured by NaSCN displacement assay (n = 8). (I) Complement activation with serum anti-NANP IgG measured by C1q deposition assay (data of one experiment; median + replicates; n = 7, LQ; n = 8, LMQ, SQ; ∗p < 0.05). (J) Complement activation of mice protected (+) vs. unprotected (−) from malaria challenge. Graph includes mice from adjuvanted groups depicted in (I) (data of one experiment; median + replicates; n = 16, protected [+]; n = 7, unprotected [−]; ∗p < 0.05, Mann-Whitney test). (K) Summary of experimental protocol for inhibition of sporozoite invasion assay (ISI). (L) ISI assay of indicated groups. Graph shows reduction of sporozoite entry into hepatocytes compared to control in percent (data of one experiment; median + replicates; n = 8; ∗∗p < 0.01). Analysis for (F), (G), (I), and (L) was performed by Kruskal-Wallis ANOVA with Dunn’s multiple comparisons. (M) ISI assay of mice protected (+) vs. unprotected (−) from malaria challenge. Graph includes mice from adjuvanted groups depicted in (L) (data of one experiment; median + replicates; n = 16, protected [+]; n = 8, unprotected [−]; ∗∗p < 0.01, Mann-Whitney test).

The R21 Ag was combined with one of four adjuvants: liposomal (L) or squalene emulsion (S) formulation containing saponin QS-21 (Q), with or without the TLR4 agonist 3D-6-acyl-PHAD (M), abbreviated as LMQ, LQ, SMQ, and SQ, respectively (Figure 1A). Employing our liver-stage mouse model for measuring sterile protection against malaria, BALB/c mice were immunized with a 3-week prime-boost regimen, followed by intravenous challenge with *Plasmodium* sporozoites (SPZ) and subsequent assessment of parasitemia in blood (Figure 1B). All adjuvanted formulations were well tolerated as no obvious adverse reactions were observed.

Remarkably, R21 with either SQ or LMQ achieved 81%–100% sterile protection (Figures 1C and S1B), while SMQ and LQ offered respectively 63% and 44% efficacy. Clinical trials of R21 with Matrix-M adjuvant indicate that IgG titers against the NANP repeat sequence in R21 correlate with protection.^12^^,^^13^ Increased IgG, IgM, and IgA titers were induced by the adjuvants. Notably, the peak titers were comparable across the four adjuvanted formulations (Figure 1D), demonstrating that quantification alone of antibody responses could not distinguish between vaccine formulations with different efficacy. Batch analysis of protected vs. unprotected mice across all groups, however, revealed a significant correlation between the highest anti-NANP IgG titers and protection (Figure 1E), in line with clinical trial data.

In measuring the IgG subclass contribution, R21 alone promoted an IgG1-dominated profile, indicating a pure T_H_2 type response (Figure 1F). In contrast, all adjuvanted groups demonstrated more mixed IgG subclass profiles, with different T_H_1/T_H_2 ratios elicited by different adjuvants. Even within the setting of T_H_2-biased BALB/c strain,^15^ LMQ was a strong T_H_1 driver, inducing the highest proportion of IgG2 and IgG3 (Figure 1F), while the second highly protective adjuvant, SQ, induced a more T_H_2-skewed response compared to LMQ (Figure 1G).

Next, we investigated the functionality of antibodies induced by different R21/adjuvant formulations seeking to (1) find any commonalities between the two most protective adjuvants and (2) detect any differences relevant to potency by comparing the adjuvants with highest and lowest efficacy. As SMQ showed intermediate efficacy (Figure 1C), we focused on LMQ and SQ as the best performing and LQ as the lowest-efficacy adjuvant in this model system. The avidity of anti-NANP IgG antibodies increased following the booster dose, with little variation across adjuvants (Figure 1H). However, both LMQ and SQ induced antibodies with higher complement activation capacity compared with LQ (Figure 1I). This also correlated with protection on a per-mouse basis, regardless of vaccine received (Figure 1J), indicating a mechanism similar to the previously observed complement-mediated merozoite neutralization.^16^ As R21 targets the pre-erythrocytic stage of malaria,^17^ an effective vaccine formulation inducing protective antibodies should elicit responses that prevent SPZ infection of the liver. We tested the capacity of the sera from vaccinated mice to block SPZ entry into hepatocytes (Figure 1K) by quantifying the inhibition of SPZ invasion (ISI) *in vitro*. R21/LMQ and R21/SQ clearly outperformed R21/LQ in eliciting antibodies that block SPZ entry into human hepatocytes (Figure 1L), strongly associating with protection (Figure 1M). This finding demonstrates the importance of antibody functionality and supports previous reports on potent SPZ neutralizing antibodies.^18^ Together, our data show that despite the divergent T_H_1/T_H_2 balance, R21/LMQ and R21/SQ both induce functionally superior antibodies over the lower efficacy adjuvant LQ.

To anticipate potential performance of these adjuvants in people, we compared the efficacy of R21/LMQ and R21/SQ with the clinically leading malaria vaccine R21/Matrix-M in our model (Figure S1A). All formulations induced 100% sterile protection (Figure S1B), with both LMQ and SQ inducing equivalent total IgG levels (Figure S1C) and complement activation as Matrix-M (Figure S1D), supporting a future clinical benefit of LMQ and SQ adjuvants. Interestingly, both Matrix-M and SQ induced similar T_H_2-biased IgG subclass distribution, in contrast to the predominantly T_H_1-biased response generated by LMQ (Figures S1E and S1F).

### Protective adjuvants LMQ and SQ trigger different innate pathways *in vitro*

**Figure 2:**
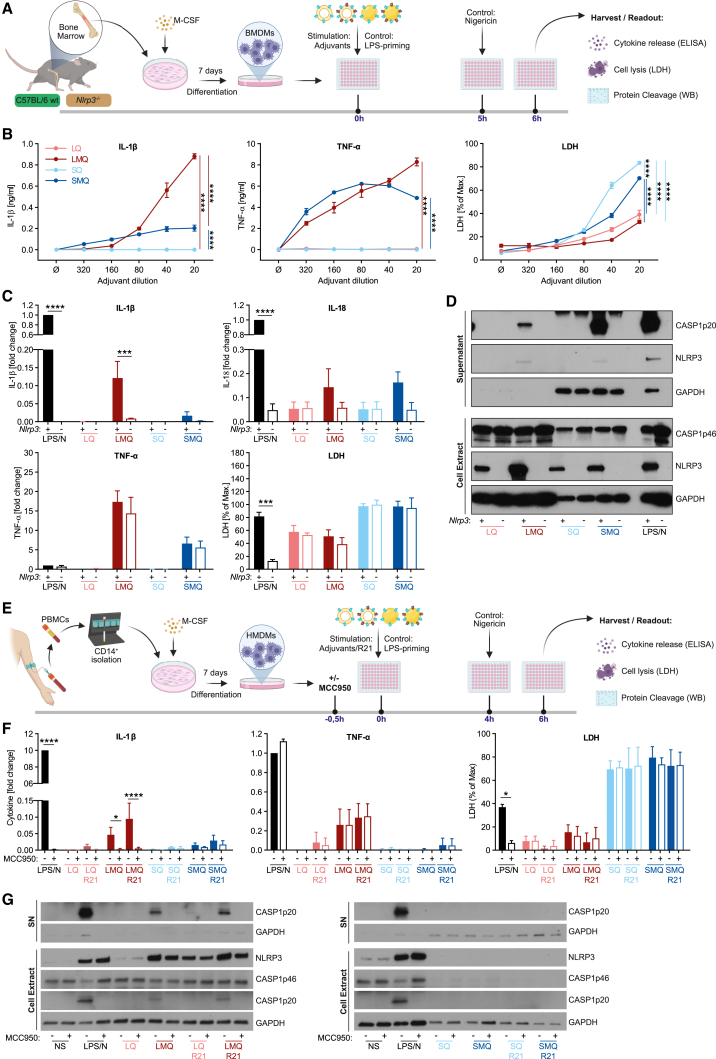
Protective adjuvants LMQ and SQ trigger different innate pathways *in vitro* (A) Summary of the experimental protocol. (B) IL-1β and TNF-α secretion in supernatants after stimulation of WT BMDMs with indicated amounts of adjuvants was measured by ELISA. LDH release was measured using a colorimetric assay and is depicted as a percentage of lysed positive control (representative data from three independent experiments; cells are stimulated in triplicates; mean ± SEM are shown). (C) BMDMs derived from C57BL/6 WT and *Nlrp3*^*−/−*^ mice were stimulated with adjuvants (1:20 dilution). Cytokines and LDH were measured as described in (B). LPS/nigericin (100 ng/mL LPS for 6 h with 5 μM nigericin for the last 1 h) stimulation was used as positive control (pooled data from three independent experiments; cytokines were normalized to LPS/nigericin control set to 1; cell death/LDH was normalized to maximal cell death control, set to 100%; cells were stimulated in triplicates; mean ± SEM are shown). (D) Representative western blots for pro-caspase-1 (p46), cleaved caspase-1 (p20), NLRP3, and GAPDH in cell lysates and supernatants from cultures of WT and *Nlrp3*^*−/−*^ BMDMs stimulated with adjuvants (1:20 dilution) or LPS/nigericin (representative data from two independent experiments). (E) Summary of the experimental protocol. (F) HMDMs were stimulated for 6 h with adjuvants (1:20 dil.) in the presence or absence of R21 (given at 1/5 of mouse dose to preserve the ratio of Ag:adjuvant given *in vivo*), in the presence or absence of NLRP3 inhibitor MCC950 (10 μM) added 0.5 h before the adjuvants. IL-1β and TNF-α secretion in supernatants was measured by ELISA. LDH release was measured using a colorimetric assay. LPS/nigericin served as a positive control (100 ng/mL LPS for 6 h with 10 μM nigericin for the last 2 h) (pooled data from three independent experiments/donors for IL-1β and LDH and two independent experiments/donors for TNF-α; cells are stimulated in triplicates; cytokines were normalized to LPS/nigericin control set to 1; cell death/LDH was normalized to maximal cell death lysis control, set to 100%; mean ± SEM are shown). (G) Representative western blots for pro-caspase-1 (p46), cleaved caspase-1 (p20), NLRP3, and GAPDH in cell lysates and supernatants from HMDMs, stimulated as in (F) (representative data from two independent experiments/donors are shown). All statistical analyses were done using two-way ANOVA with Bonferroni’s correction for multiple comparisons; ∗p < 0.05, ∗∗p < 0.01, ∗∗∗p < 0.001, ∗∗∗∗p < 0.0001. In (B), significant adjuvant effect is reported. In (C) and (F), significant genotype effect is reported.

To explore the MoA of the two most protective adjuvants at the molecular level, we focused on the inflammasome pathway as a key innate sensor,^19^ known to mediate adjuvanticity of QS-21 in the presence of TLR4 ligands.^20^^,^^21^ *In vitro* response to the adjuvants was measured in bone marrow-derived macrophages (BMDMs) from BALB/c wild-type (WT) (Figures S2A and S2B) and C57BL/6 WT and *Nlrp3*^*−/−*^ mice (Figure 2A). Unlike SQ, LMQ stimulated NLRP3 expression, NLRP3-induced caspase-1 activation, and robust secretion of NLRP3-dependent innate cytokines IL-1β and IL-18, all diminished in *Nlrp3*^*−/−*^ cells (Figures 2B–2D). Additionally, LMQ activated the TLR4 pathway and elicited secretion of the TLR4-induced, NLRP3-independent, cytokine TNF-α. Substantial levels of cell death, as indicated by the release of cytosolic lactate dehydrogenase (LDH), were observed with the highest doses of the emulsion-based (SQ, SMQ) and not with the liposomal (LQ, LMQ) adjuvants (Figures 2B, 2C, and S2B). Surprisingly, SMQ, which can also induce expression and activation of NLRP3 (Figure 2D), elicited very low levels of IL-1β in comparison with LMQ (Figure 2C). We postulate that the cell lysis observed in response to high doses of emulsion adjuvants *in vitro* likely prevented SMQ from matching the cytokine levels induced by LMQ (Figures 2B, 2C, and S2B). Thus, changing the adjuvant base formulation from emulsion (SMQ) to liposomal (LMQ), while retaining the same immunostimulatory targets (NLRP3 and TLR4), may limit the cell death levels *in vitro* along with enhancing protection *in vivo*.

These findings were corroborated in human blood monocyte-derived macrophages (HMDMs, Figures 2E–2G). We found that LMQ induced NLRP3 protein expression (Figure 2G) and NLRP3-dependent secretion of IL-1β that was abrogated in the presence of the NLRP3 inhibitor MCC950 (Figure 2F). LMQ also stimulated NLRP3-induced caspase-1 activation (Figure 2G), as well as the secretion of the TLR4-induced but NLRP3-independent cytokine TNF-α (Figure 2F).^22^ As identified with mouse BMDMs, both emulsion adjuvants (SQ and SMQ) also elicited substantial levels of cell death in HMDMs at the tested doses, as indicated by the release of cytosolic LDH (Figure 2F), which almost completely prevented SMQ from matching the cytokine levels and caspase-1 activation induced by LMQ (Figures 2F and 2G). Addition of R21 did not change the signaling properties of adjuvants or their ability to activate the NLRP3 inflammasome, although it did show some boosting effect on NLRP3 protein expression (Figures 2F and 2G).

Downstream of NLRP3 activation, cytokine release and cell death responses are mediated by pore-forming proteins—gasdermins (GSDMs), in particular gasdermin D (GSDMD).^23^^,^^24^^,^^25^ Using BMDMs derived from WT or *Gsdmd*^*−/−*^ mice, we found that both LMQ and SMQ induced cleavage of GSDMD into its active membrane pore-forming fragment, and NLRP3 induced IL-1β release that was mediated by GSDMD (Figures S3A−S3C). As expected, the release of the NLRP3-independent cytokine TNF-α was independent of GSDMD (Figure S3B). However, GSDMD deficiency did not fully abrogate IL-1β secretion by LMQ or SMQ, suggesting an additional IL-1β release mechanism downstream of NLRP3.^26^ In some settings, gasdermin E (GSDME) pores can provide such release,^27^^,^^28^ but we found that GSDME deficiency did not affect LMQ- or SMQ-induced caspase-1 cleavage or IL-1β secretion (Figures S3D−S3E), indicating no role of GSDME in mediating immune responses to these adjuvants *in vitro*. Finally, cell death caused by emulsion adjuvants (SQ and SMQ) was independent of both GSDMs, indicating an inflammasome-independent lytic response (Figures S3B and S3D).

In conclusion, our two most protective vaccine formulations, R21/LMQ and R21/SQ, triggered very different innate sensing mechanisms in macrophages *in vitro* with LMQ, but not SQ, activating the NLRP3 inflammasome and SQ, but not LMQ, activating an inflammasome-independent cell lytic response.

### Protective adjuvants LMQ and SQ trigger disparate innate responses *in vivo*

**Figure 3:**
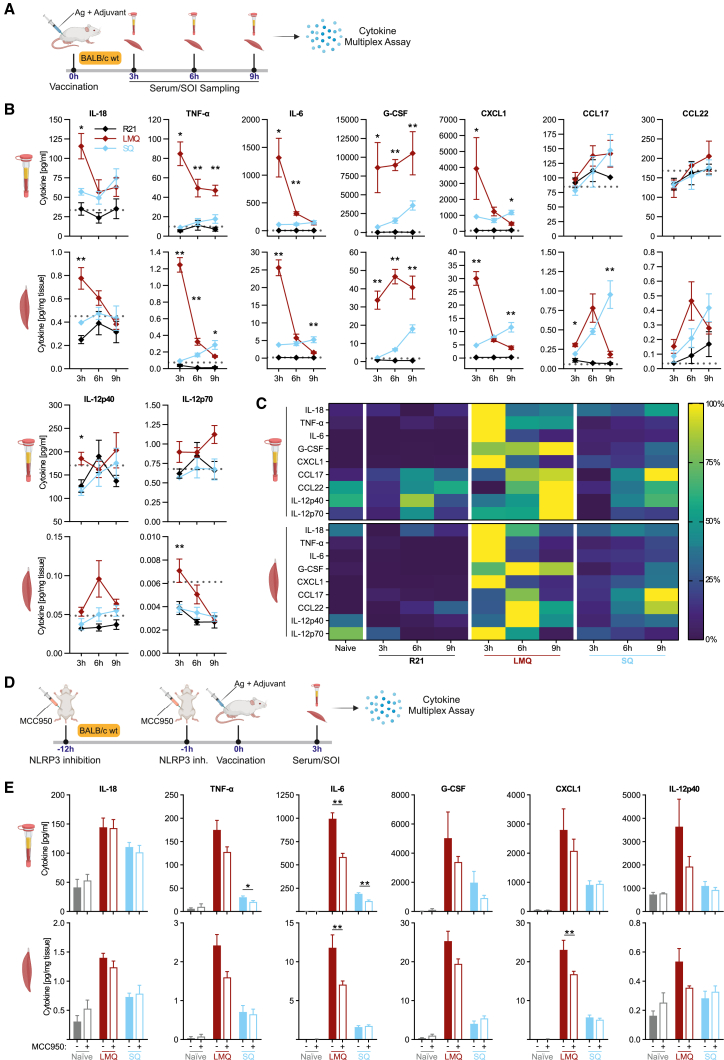
Protective adjuvants LMQ and SQ trigger disparate innate responses *in vivo* (A) Summary of experimental protocol. Site of injection (SOI). (B) Indicated cytokines were measured by LEGENDplex assay (dashed line = mean of naive group; mean ± SEM; n = 5; ∗p < 0.05, ∗∗p < 0.01, Mann-Whitney test per time point between SQ/LMQ). (C) Heatmap of indicated cytokines, measured as described in (B). (D) Summary of experimental protocol. (E) Indicated cytokines were measured by LEGENDplex assay (mean ± SEM; n = 6; ∗p < 0.05, ∗∗p < 0.01, Mann-Whitney test per adjuvant between ± MCC950).

We next investigated the *in vivo* innate response to the two most protective vaccine formulations, R21/LMQ and R21/SQ, by mapping the kinetics of secreted cytokines and chemokines in the serum and at the site of vaccination (Figures 3A–3C). In line with the *in vitro* data, LMQ triggered a strong and immediate secretion of IL-18 and many TLR4- and NF-κB-induced inflammatory mediators, such as TNF-α, IL-6, G-CSF, CXCL1, CCL17, CCL22, and IL-12p40.^29^^,^^30^^,^^31^^,^^32^^,^^33^^,^^34^^,^^35^ In contrast, SQ induced generally lower and delayed secretion of IL-18 and most of the other tested cytokines/chemokines, with the exception of a late release of CCL17. As SQ does not contain TLR4 ligands that would contribute to direct NLRP3 priming, we propose that SQ *in vivo* may elicit a low-level release of DAMPs, such as HMGB1 or IL-1α, that facilitate a weaker, delayed, systemic innate immune activation.

To evaluate the role of NLRP3-derived cytokines IL-18 and IL-1β in the early innate response to our vaccine formulations, we blocked NLRP3 function by injecting mice intraperitoneally with NLRP3 inhibitor MCC950 prior to vaccination (Figure 3D). This did not affect IL-18 secretion in response to either R21/LMQ or R21/SQ at 3 h post-immunization (Figure 3E); however, it did diminish the secretion of downstream targets of NF-κB, such as IL-6, in response to R21/LMQ. The abrogating effect of MCC950 was substantially stronger in response to LMQ than SQ. We propose that, in the context of R21/LMQ *in vivo*, even low IL-1β levels are sufficient to amplify NF-κB activation via IL1R1,^36^ synergizing with TLR4 triggering by LMQ and boosting the systemic secretion of multiple pro-inflammatory cytokines.

Taken together, *in vitro* and *in vivo* studies of the immune signaling by R21/LMQ and R21/SQ reveal considerable differences in the quality, magnitude, and kinetics of the innate response generated by the two vaccine formulations.

### R21/LMQ and R21/SQ induce disparate CD4^+^ T_H_1 and T_H_2 responses

Different innate cytokine and chemokine profiles resulting from vaccination typically skew the adaptive T cell helper response toward T_H_1 or T_H_2, eventually resulting in distinct antibody switching. As noted earlier, R21/LMQ and R21/SQ induced different IgG subclass profiles in BALB/c mice, suggesting divergent T_H_1/T_H_2 bias (Figure 1G). In this context, the NLRP3/caspase-1/IL-18 axis has been demonstrated as a crucial decision node.^37^^,^^38^ Immunization with R21/LMQ induces secretion of IL-18 and some IL-12 (Figure 3B), a cytokine combination that induces IFN-γ, a strong driver of T_H_1 cellular response.^39^^,^^40^^,^^41^ In contrast, as we observed with R21/SQ, it has been reported that IL-18 in the absence of IL-12 drives skewing toward T_H_2.^38^^,^^42^

**Figure 4:**
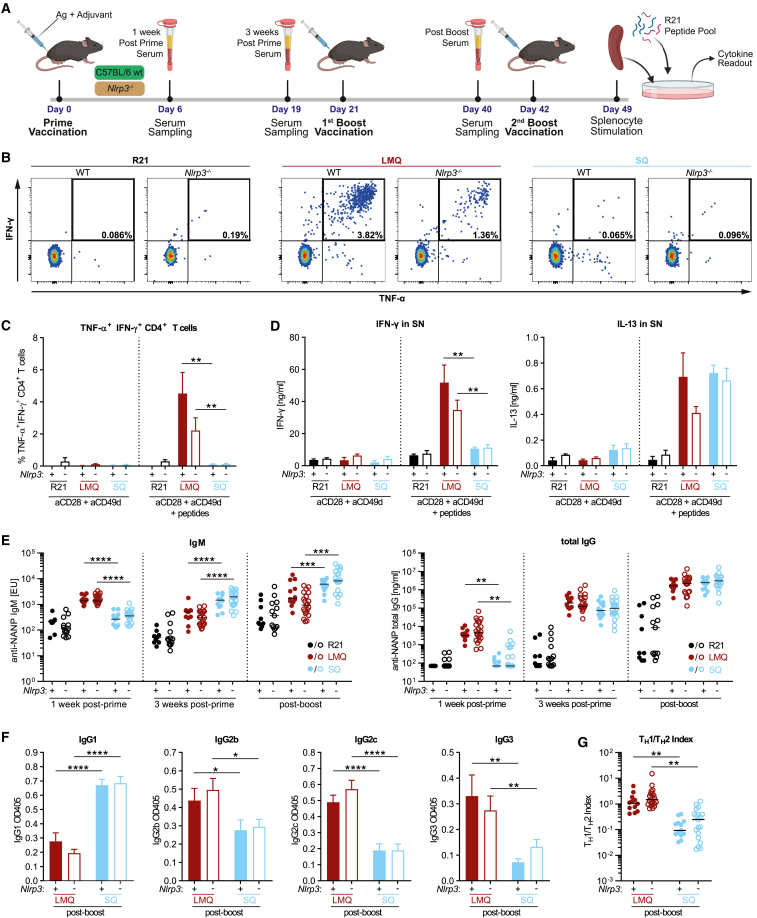
R21/LMQ and R21/SQ induce disparate CD4^+^ T cell responses (A) Summary of the experimental protocol. C57BL/6 mice require three vaccine doses to develop full response against R21.^43^ (B) Representative FACS plots of R21 peptide pool stimulated splenocytes from mice with indicated genotype and vaccine adjuvant used. Graphs show IFN-γ and TNF-α double-positive CD4^+^ T cells. Cells were pre-gated on live single cells, CD19^−^, CD3^+^, and CD8^−^. (B and C) Representative data from two experiments; mean ± SEM; n = 3 for WT/R21, WT/LMQ, and WT/SQ; n = 4 for *Nlrp3*^*−/−*^/R21; n = 5 for *Nlrp3*^*−/−*^/LMQ and *Nlrp3*^*−/−*^/SQ. (C) Quantification and summary of data represented in (B). (D) IFN-γ and IL-13 in supernatants of R21 peptide pool stimulated splenocytes from mice with indicated genotype and vaccine adjuvant used, as measured by ELISA. Representative data from two experiments. (E and F) Pooled data from three experiments; median + replicates; n = 9, WT/R21; n = 13, *Nlrp3*^*−/−*^/R21; n = 12, WT/LMQ; n = 22, *Nlrp3*^*−/−*^/LMQ; n = 13, WT/SQ; n = 20, *Nlrp3*^*−/−*^/SQ. (E) Anti-NANP serum antibody titers of indicated isotypes. C57BL/6 WT or *Nlrp3*^*−/−*^ mice were vaccinated and sampled as indicated in (A). Titers were assessed by ELISA. (F) Detection of anti-NANP IgG subclasses by proportional ELISA. Each sample was diluted and normalized to 80 ng/mL of total IgG. Graphs show optical density against indicated IgG subclasses. Pooled data from three independent experiments; mean ± SEM; n = 12, WT/LMQ; n = 22, *Nlrp3*^*−/−*^/LMQ; n = 13, WT/SQ; n = 20, *Nlrp3*^*−/−*^/SQ. (G) T_H_1/T_H_2 index of adjuvant-induced subclass patterns. Index was calculated as ([IgG2c + IgG3]/2)/IgG1.^44^ Increased T_H_1/T_H_2 index indicates T_H_1-skewed immune response. Pooled data from three independent experiments; median + replicates; n = 12, WT/LMQ; n = 21, *Nlrp3*^*−/−*^/LMQ; n = 13, WT/SQ; n = 19, *Nlrp3*^*−/−*^/SQ; all statistical analyses were done using two-way ANOVA of adjuvanted groups with Bonferroni’s multiple comparisons; ∗∗p < 0.01, ∗∗∗p < 0.001, ∗∗∗∗p < 0.0001.

To evaluate how congenital NLRP3 deficiency affects the immune response to R21/LMQ and R21/SQ, we immunized WT and *Nlrp3*^*−/−*^ C57BL/6 mice (Figure 4A). Restimulated splenocytes from R21/LMQ vaccinated mice displayed a mixed T_H_1/T_H_2 response, with T_H_1-type IFN-γ^+^/TNF-α^+^ CD4^+^ T cells and secretion of T_H_2-type cytokine IL-13, both partially reduced in *Nlrp3*-deficient mice. Immunization with R21/SQ mainly induced T_H_2 response IL-13 secretion, which was not affected by lack of *Nlrp3* (Figures 4B–4D).

Despite the reduced CD4^+^ T cell activation in the absence of NLRP3, antibody production was not affected, with equal IgM and IgG titers detected between WT and *Nlrp3*^*−/−*^ mice in both R21/LMQ and R21/SQ groups (Figure 4E). Interestingly, we observed a difference in the kinetics of antibody generation and isotype switching: 1 week post-first immunization, mice vaccinated with R21/LMQ had higher levels of IgM and had already switched to IgG compared with mice receiving R21/SQ. 5 weeks later (after the second immunization), both vaccine groups reached similar IgG titers (Figure 4E).

Our results indicate that the early fast and strong R21/LMQ-induced T_H_1-type innate cytokine profile (Figure 3) mirrors a faster humoral response in comparison with R21/SQ. In this setting, LMQ promotes switching to T_H_1-type IgG profile dominated by IgG2 and IgG3, whereas SQ induces a T_H_2-type skewed IgG1 response (Figures 4F and 4G). In the context of LMQ, NLRP3 synergizes with TLR4 activation in eliciting early innate immunity and in shaping the CD4^+^ T helper response. *In vivo*, NLRP3 deficiency can be overcome and compensated for systemically to allow the generation of a protective B cell response.

## Discussion

The clear value of safe and effective adjuvants and the necessity of their use with an ever-increasing list of antigens have intensified efforts to better understand their MoA. Access to multiple effective immunization approaches would also de-risk large-scale vaccine production and safeguard against supply and logistics issues, especially in a future pandemic scenario.^45^^,^^46^

Here, we focus on two open access adjuvants, LMQ and SQ, which in combination with R21 malaria antigen offer >80% protection against lethal challenge in mice. Such high efficacy is comparable with Novavax’s Matrix-M and GSK’s AS01B,^17^^,^^43^ and it is potentially superior to CSL’s MF59^17^ or a *Pf*CSP-based mRNA vaccine,^47^ but this remains to be formally tested. For pre-erythrocytic malaria vaccines such as R21, the induction of a robust humoral response is crucial to prevent SPZ entry into hepatocytes.^48^ Nevertheless, and in line with studies in humans,^49^ we found that the total antibody titers post-vaccination do not predict vaccine efficacy. Rather, LMQ and SQ promoted the generation of functionally superior antibodies, with enhanced activation of the complement system and stronger neutralization capacity. This demonstrates that changes in adjuvant formulation may increase vaccine efficacy through improved functionality of the humoral response independent of its magnitude.

We discovered that R21/LMQ and R21/SQ provide protection against malaria by distinct and divergent immune responses. While R21 alone induces a pure T_H_2-type immunity, the addition of adjuvants initiates different innate triggers of the adaptive response, with R21/LMQ inducing a far more T_H_1-skewed response than R21/SQ. This dichotomy is reflected in both the initial early cytokine and chemokine responses and in the antibody subclass composition and the CD4^+^ T cell responses several weeks later. The origin of the divergent instruction may lie in the different innate profiles initiated by LMQ and SQ: LMQ, through a combined engagement of NLRP3 and TLR4 pathways, stimulates enhanced and rapid IL-18 and IL-12 secretion, a strong T_H_1 driver,^38^^,^^39^^,^^41^ whereas SQ-induced delayed IL-18, in the absence of detectable IL-12, promotes T_H_2 polarization.^38^^,^^42^ Systemically, *in vivo*, QS-21 in SQ has the potential to activate NLRP3,^20^^,^^21^ even without a TLR4 agonist. This is likely achieved through the release of DAMPs (as observed *in vitro*), facilitating a delayed, lower-grade activation of the inflammasome for IL-18 secretion, and toward protective, more T_H_2-skewed immunity. In addition, at the injection site, SQ induces sustained levels of CCL17 and CCL22, two chemo-attractants binding CCR4 expressed by T_H_2-type T cells.^50^

Through these different axes, R21/LMQ and R21/SQ set the groundwork for the ensuing adaptive immunity within hours post-immunization, retaining the T_H_1/T_H_2 bias throughout the humoral response over several weeks. The T_H_1-driven immune profile induced by LMQ is in line with the clinically deployed AS01 adjuvant, which in comparison with the squalene emulsion MF59 promotes stronger IFN-γ^+^ and TNF-α^+^ CD4^+^ T cell responses and IgG2a production.^17^^,^^51^^,^^52^ Conversely, the mechanism underpinning SQ adjuvanticity opens exciting routes for future vaccine development by offering equally good protection, most likely through a DAMP-dependent pathway, in the absence of direct NLRP3 activation.

Surprising findings were generated with SMQ and LMQ, which contain the same immuno-stimulating components (TLR4 ligand and QS-21), in a different formulation (emulsion vs. liposomal). *In vitro*, SMQ was more lytic to macrophages than LMQ, which limited its ability to induce TNF-α and IL-1β and offered lower protection against malaria. Therefore, by changing the adjuvant formulation and dose, we could fine-tune levels of cell death *in vitro*. Our findings support the model put forward by Kagan and colleagues, which proposes that inflammasome-activating compounds that do not elicit strong cell death would generate more potent vaccine formulations.^53^

The contribution of inflammasome signaling to the eventual adaptive responses is still under debate,^19^ and the level of inflammasome involvement in adjuvant activity may depend on the vaccine antigen. Although we did not detect CD8^+^ T cell responses to the R21 antigen, QS-21 in combination with other antigens can elicit CD8^+^ T cells that can be boosted by the inflammasome pathway.^20^ Furthermore, SQ combined with MERS-CoV RBD antigen induced robust CD4^+^ and CD8^+^ T cell Th1-type response, comparable with LMQ.^54^ These data suggest that the ability of adjuvants to strongly activate NLRP3 may be a valuable mechanism for vaccines targeting CD8^+^ T cell immunity, such as HIV vaccine candidates.

In conclusion, we explored a portfolio of potent open access adjuvants that enhance the immunogenicity and efficacy of the highly successful malaria vaccine antigen R21. Combining different adjuvant formulations with a single antigen/vaccine, along with a stringent *in vivo* challenge model and multiple strains of mice, and elucidation of APC activation pathways accompanied with cellular and humoral responses, allows biological discrimination of various adjuvant effects with the potential to inform future clinical use and mechanistically guided vaccine design.

### Limitations of the study

Functionally, we did not test whether memory responses or the Ab repertoire may vary when different innate pathways are engaged to prime adaptive immunity; this is the subject of the follow-up work. We also did not have equal freedom to operate with Matrix-M; it was only used as a benchmark in key *in vivo* efficacy experiments. Further, all adjuvants were used at a single dose *in vivo*, and more detailed dose-response studies would likely be needed. Mechanistically, this study did not systemically address whether the engaged innate pathways may differ in other key cells that would sense these adjuvants *in vivo*, such as dendritic cells or neutrophils. This remains to be tested.

## STAR★Methods

### Key resources table

**Table:** 

REAGENT or RESOURCE	SOURCE	IDENTIFIER
**Antibodies**		

Fc-specific goat anti-mouse IgG-AP	Sigma-Aldrich	Cat#A1418; RRID:AB_257932
Goat Anti-Mouse IgM mu chain-AP	Abcam	Cat#ab98672; RRID:AB_10674742
Goat Anti-Mouse IgA-AP	Southern Biotech	Cat#1040-04; RRID:AB_2794372
2A10 (anti-NANP)	MR4	Cat#MRA-183A
Anti-mouse IgG1-AP	Southern Biotech	Cat#1071-04; RRID:AB_2794425
Anti-mouse IgG2a-AP	Southern Biotech	Cat#1081-04; RRID:AB_2794494
Anti-mouse IgG2b-AP	Southern Biotech	Cat#1091-04 RRID:AB_2794541
Anti-mouse IgG2c-AP	Southern Biotech	Cat#1078-04; RRID:AB_2794461
Anti-mouse IgG3-AP	Abcam	Cat#ab98705; RRID:AB_10674160
Goat anti-C1q-FITC	Abcam	Cat#ab182940 - discontinued. Suggested replacement: ab4223; RRID: AB_304387
Donkey anti-goat-IgG-AP	Abcam	Cat#ab6886; RRID:AB_954626
Mouse caspase-1 (casper-1) antibody	AdipoGen	Cat#AG-20B-0042-C100; RRID: AB_2490248
Rabbit monoclonal GAPDH (14C10) antibody	CST	Cat#2118S; RRID: AB_561053
Mouse NLRP3 (Cryo-2) antibody	Caltag Medsystems	Cat#AG-20B-0014-C100; RRID:AB_2885199
Mouse tubulin antibody	Sigma-Aldrich	Cat#T5168; RRID: AB_477579
Human Caspase-1 (D7F10) Rabbit mAb	Cell Signaling	Cat#3866; RRID:AB_2069051
Recombinant Anti-GSDMD antibody [EPR19828]	Abcam	Cat#ab209845; RRID:AB_2783550
Recombinant Anti-DFNA5/GSDME antibody [EPR19859] - N-terminal	Abcam	Cat#ab215191; RRID:AB_2737000
Anti-mouse CD28 Antibody	Biolegend	Cat#102112; RRID:AB_312877
Ultra-LEAF™ Purified anti-mouse CD49d Antibody	Biolegend	Cat#103709; RRID:AB_2832316
FITC anti-mouse TNF-α Antibody	Biolegend	Cat#506304; RRID:AB_315425
PE anti-mouse IFN-γ Antibody	Biolegend	Cat#505808; RRID:AB_315402
CD3-PECy7 (anti-mouse)	Biolegend	Cat#100220; RRID:AB_1732057
CD19-APCCy7 (anti-mouse)	Biolegend	Cat#115530; RRID: AB_830707
CD4-APC (anti-mouse)	Biolegend	Cat#100412; RRID:AB_312697
BV605-CD8 (anti-mouse)	Biolegend	Cat#100743; RRID:AB_2561352

**Biological samples**		

Peripheral blood from healthy donors	NHS Oxford blood bank	N/A
Murine spleens, muscle, and peripheral blood	This paper	N/A

**Chemicals, peptides, and recombinant proteins**		

Peptide CSP1: MMAPDPNANPNANPN	Mimotopes	N/A
Peptide CSP2: NANPNANPNANPNAN	Mimotopes	N/A
Peptide CSP3: DPNANPNANPNKNNQ	Mimotopes	N/A
Peptide CSP4: NPNANPNKNNQGNGQ	Mimotopes	N/A
Peptide CSP5: NPNKNNQGNGQGHNM	Mimotopes	N/A
Peptide CSP6: NNQGNGQGHNMPNDP	Mimotopes	N/A
Peptide CSP7: NGQGHNMPNDPNRNV	Mimotopes	N/A
Peptide CSP8: HNMPNDPNRNVDENA	Mimotopes	N/A
Peptide CSP9: NDPNRNVDENANANS	Mimotopes	N/A
Peptide CSP10: RNVDENANANSAVKN	Mimotopes	N/A
Peptide CSP11: ENANANSAVKNNNNE	Mimotopes	N/A
Peptide CSP12: ANSAVKNNNNEEPSD	Mimotopes	N/A
Peptide CSP13: VKNNNNEEPSDKHIK	Mimotopes	N/A
Peptide CSP14: NNEEPSDKHIKEYLN	Mimotopes	N/A
Peptide CSP15: PSDKHIKEYLNKIQN	Mimotopes	N/A
Peptide CSP16: HIKEYLNKIQNSLST	Mimotopes	N/A
Peptide CSP17: YLNKIQNSLSTEWSP	Mimotopes	N/A
Peptide CSP18: IQNSLSTEWSPCSVT	Mimotopes	N/A
Peptide CSP19: LSTEWSPCSVTCGNG	Mimotopes	N/A
Peptide CSP20: WSPCSVTCGNGIQVR	Mimotopes	N/A
Peptide CSP21: SVTCGNGIQVRIKPG	Mimotopes	N/A
Peptide CSP22: GNGIQVRIKPGSANK	Mimotopes	N/A
Peptide CSP23: QVRIKPGSANKPKDE	Mimotopes	N/A
Peptide CSP24: KPGSANKPKDELDYA	Mimotopes	N/A
Peptide CSP25: ANKPKDELDYANDIE	Mimotopes	N/A
Peptide CSP26: KDELDYANDIEKKIC	Mimotopes	N/A
Peptide CSP27: DYANDIEKKICKMEK	Mimotopes	N/A
Peptide CSP28: DIEKKICKMEKCSSV	Mimotopes	N/A
Peptide CSP29: KICKMEKCSSVFNVV	Mimotopes	N/A
Peptide CSP30: MEKCSSVFNVVNSSI	Mimotopes	N/A
Peptide CSP31: KCSSVFNVVNSSIGL	Mimotopes	N/A
Peptide for anti-NANP ELISAs: NANPNANPNANPNANPNANPNANPC	Mimotopes	N/A
CP-456773 (MCC950)	Sigma-Aldrich	Cat#PZ0280-25MG CAS:256373-96-3
M-CSF	Immunotools	Cat#11343118
Cholesterol	Merck-Sigma, USA	Cat#C1231 CAS:57-88-5
QS21 solution (GMP grade)	Desert King International, USA	N/A
Synthetic TLR4 ligand 3D-6-acyl-PHAD (3D6AP)	Merck-Avanti, USA	Cat#770050
1,2-dioleoyl-*sn*-glycero-3-phosphocholine (DOPC)	Merck-Avanti, USA	Cat#730375
Pierce™ Diethanolamine Substrate Buffer	Thermo Fisher	Cat#34064
4-Nitrophenyl phosphate disodium salt hexahydrate	Sigma-Aldrich	Cat# N2765-100TAB CAS:333338-18-4
C1q (human)	Sigma-Aldrich	Cat#C1740 CAS:80295-33-6
Protease inhibitor	Roche	Cat#11873580001
TrypLE™ Express Enzyme	Life Technologies	Cat#12605010
DAPI	Sigma-Aldrich	Cat#D9542 CAS:28718-90-3
*E.Coli* K12 ultrapure LPS	Invivogen	Cat#tlrl-peklps
Nigericin	Sigma-Aldrich	Cat#N7143-5MG; CAS:28643-80-3
CaptureSelect™ C-tagXL Affinity Matrix	Thermo Fisher	Cat#2943072005

**Critical commercial assays**		

LEGENDplex™ macrophage/microglia multi-analyte flow assay kit	Biolegend	Cat#740846
CytoTox 96® Non-Radioactive Cytotoxicity Assay	Promega	Cat#G1780
IL-1 beta Mouse ELISA Kit	Thermo Fisher	Cat#88-7013-77
TNF alpha Mouse ELISA Kit	Thermo Fisher	Cat#88-7324-77
IL-18 Mouse ELISA Kit	Thermo Fisher	Cat#BMS618-3
IL-1 beta human ELISA Kit	Thermo Fisher	Cat#88-7261-77
TNF alpha Human ELISA Kit	Thermo Fisher	Cat#88-7346-88
IL-13 Mouse ELISA Kit	Thermo Fisher	Cat#88-7137-88
IFN gamma Mouse ELISA Kit	Thermo Fisher	Cat#88-7314-88
MagniSort™ Human CD14 Positive Selection Kit	Thermo Fisher	Cat#8802-6834-74

**Deposited data**		

Original Western blot images	Mendeley	https://doi.org/10.17632/2rfymh6b78.1

**Experimental models: Cell lines**		

Huh7, Hepatocyte cell line	Dr. Chris J. Janse, University of Leiden	N/A

**Experimental models: Organisms/strains**		

Mouse: C57BL/6J	Jackson Laboratories	RRID:IMSR_JAX:000664
Mouse: C57BL/6N	Jackson Laboratories	RRID:IMSR_JAX:005304
Mouse: B6.129S6-*Nlrp3*^*tm1Bhk*^/J	Jackson Laboratories	RRID:IMSR_JAX:021302
Mouse: C57BL/6J-*Gsdmd*^*em1Vnce*^/J	Jackson Laboratories	RRID:IMSR_JAX:032663
Mouse: C57BL/6N-*Gsdme*^*em1Fsha*^/J	Jackson Laboratories	RRID:IMSR_JAX:03241
Mouse: BALB/cOlaHsd	Envigo, UK	RRID:IMSR_ENV:HSD-162
*P. berghei* transgenic parasites: (*PfCSP(r)PbCSP + GFP::Luc@Pbeef1a_230p*)	The Jenner Institute^43^	N/A
*Anopheles stephensi* mosquitoes	Prof. Robert Sinden, Imperial College London	N/A
*Pichia pastoris*: PichiaPink™ Strain 1: ade2	Invitrogen™ Fisher Scientific	Cat#A11154

**Recombinant DNA**		

PichiaPink™ Expression System	Invitrogen™ Fisher Scientific	Cat#10329653

**Software and algorithms**		

BD FACSDiva™ Software	https://www.bdbiosciences.com	RRID:SCR_001456
FlowJo	https://www.flowjo.com	RRID: SCR_008520
GraphPad Prism	https://www.graphpad.com	RRID: SCR_002798
LEGENDplex™ Data Analysis Software Suite	https://www.biolegend.com	N/A

**Other**		

GentleMACS™ M Tubes	Miltenyi Biotec	Cat#130-093-236
GentleMACS™ Octo Dissociator	Miltenyi Biotec	Cat#130-096-427
XK16 column	GE Healthcare Life Sciences	Cat#GE28-9889-37
HiLoad® 16/600 Superdex® 200 pg	GE Healthcare Life Sciences	Cat#GE28-9893-35

### Resource availability

#### Lead contact

All inquiries should be addressed to the lead contact, Anita Milicic (anita.milicic@ndm.ox.ac.uk).

#### Materials availability

The R21 antigen can be made available through a Materials Transfer Agreement. Access can be requested through the lead contact Anita Milicic. VFI adjuvants can be made available through contact via VFI website (https://www.vaccineformulationinstitute.org/). Further information and requests for reagents should be directed to and will be fulfilled by Anita Milicic and/or Jelena Bezbradica.

### Experimental model and subject details

#### Mice

For all experiments, animals were maintained at the Wellcome Center for Human Genetics or the Kennedy Institute of Rheumatology, University of Oxford. Animals were housed under Specific Pathogen Free (SPF) conditions and in accordance with the recommendations of the UK Animals (Scientific Procedures) Act 1986 and ARRIVE guidelines. Protocols were approved by the University of Oxford Animal Care and Ethical Review Committee for use under Project Licenses P9804B4F1, PP0984913, or P464EC8CB granted by the UK Home Office.

NLRP3 KO mice (B6.129S6-*Nlrp3*^*tm1Bhk*^/J), GSDMD KO mice (C57BL/6J-*Gsdmd*^*em1Vnce*^/J), GSDME KO mice (C57BL/6N-*Gsdme*^*em1Fsha*^/J mice) and appropriate C57BL/6J and C57BL/6N controls were obtained from Jackson Laboratories. In-house bred C57BL/6J mice were used as a source of bone marrow cells to generate bone marrow-derived macrophages *in vitro*. BALB/c mice (BALB/cOlaHsd) were purchased from Envigo, UK. BALB/c mice were all female, all other animals used were mixed sex.

#### Transgenic parasite and sporozoite production

The transgenic parasites were generated as previously described using the ‘gene insertion/marker out’ technology.^55^ *P. berghei* transgenic parasites contain an additional copy of the *P. falciparum* CSP gene inserted at the 230p locus under the control of the *P. berghei* UIS4 promoter (*PfCSP(r)PbCSP + GFP::Luc@Pbeef1a_230p*).^43^

Transgenic sporozoites were produced as previously described.^17^ Starved female *Anopheles stephensi* mosquitoes were fed on BALB/c mice infected with the transgenic parasites for approximately 10 min. The mosquitoes were maintained on Fructose/PABA solution at 19–21°C in a humidified incubator on 12-h day-night cycle. Approximately 21 days after feeding, the mosquitoes were dissected and the salivary glands removed and placed in RPMI-1640. The glands were disrupted to release the sporozoites with a tissue homogeniser and the sporozoites counted using a haemocytometer.

#### Bone marrow-derived macrophages (BMDMs)

BMDMs were generated by differentiating them from total mouse bone marrow for 7 days with recombinant M-CSF (50 ng/mL, Immunotools, 11343118). Cells were cultured in complete macrophage medium (CMM) consisting of RPMI (Fisher, 21875091) with 10% FBS (Gibco, certified low endotoxin, 1600044), 1x Pen/Strep/Glutamine (Fisher, 10378016), and 10–25 mM HEPES (Fischer, 15630056) at 37°C with 5% CO_2_ and supplemented with fresh media containing 50 ng/mL M-CSF on day 5. After day 7 of differentiation, cells were replated (100,000 cells per well in flat bottom 96-well plate in 100 μL CMM) and on day 8 cells were stimulated for inflammasome experiments.

#### Human monocyte-derived macrophages (HMDMs)

Peripheral blood mononuclear cells were isolated using Ficoll gradient from healthy donors from NHS Oxford blood bank (REC approval 11/H0711/7). CD14^+^ magnetic beads (Biolegend, 8802-6834-74) were used to positively select monocytes. CD14^+^ cells were differentiated into macrophages by culturing them for 7 days with M-CSF (100 ng/mL, Immunotools, 11343118). Cells were cultured in RPMI (Fisher, 21875091) with 10% FBS (Gibco, certified low endotoxin, 1600044) and 1x Pen/Strep/Glutamine (Fisher, 10378016) at 37°C with 5% CO_2_ and supplemented with fresh media containing 100 ng/mL M-CSF on day 5. After day 7 of differentiation, cells were replated (70,000 cells per well in flat bottom 96-well plate in 100 μL CMM) and on day 8 cells were stimulated for inflammasome experiments.

### Method details

#### Adjuvants and formulations

Adjuvants used in this study (SQ, SMQ, LQ and LMQ) were manufactured at the Vaccine Formulation Institute as described previously.^54^ Briefly, SQ adjuvant was prepared by combining squalene-in-water emulsion containing cholesterol (Merck-Sigma, USA) with QS-21 in solution (Desert King International, USA). SMQ was prepared in a similar fashion with the emulsion containing both cholesterol and synthetic TLR4 ligand 3D-6-acyl-PHAD (3D6AP) (Merck-Avanti, USA). LQ adjuvant was prepared by combining liposomes composed of 1,2-dioleoyl-*sn*-glycero-3-phosphocholine (DOPC, Merck-Avanti, USA) and cholesterol with QS-21, and LMQ adjuvant was prepared as LQ but with incorporation of 3D6AP during liposome preparation. R21 antigen stability at 1:1 volume ratio was documented with all adjuvants and checked for adjuvant physicochemical characteristics including pH, particle size, polydispersity, zeta potential and composition including DOPC, cholesterol, squalene, 3D6AP and QS-21 content. Each injectable dose of LQ, SQ, LMQ and SMQ contained 5 μg of QS-21 saponin and and/or 2 μg of the TLR4 agonist 3D6AP. Macrophages *in vitro* were stimulated with adjuvants starting with a 1:20 dilution, followed by serial 1:2 dilutions. At the highest dose this translates to 1 μg of QS-21 and 0.4 μg TLR4 agonist per well in 100 μL media.

The virus-like particle R21 vaccine (C-tagged) was generated as described previously.^43^ The gene for expression of R21c was cloned from the R21 expression plasmid^17^ into the PichiaPink expression plasmid pPink-HC using a reverse primer containing the C-Tag. Linearised plasmid DNA was transformed into electrocompetent PichiaPink strain 1 cells. Yeast was grown as described previously.^17^ Protein expression was induced with 1% methanol once per day. Cells were harvested by centrifugation and lysed in the presence of benzonase and detergent using glass beads. C-tagged proteins were purified from the lysates over a C-Tag affinity column prepared with 5 mL CaptureSelect C-tag Affinity Matrix (Thermo Fisher) packed into an XK16 column (GE Healthcare Life Sciences) with 2 M MgCl_2_ elution buffer. VLPs were further purified by size exclusion chromatography over a HiLoad 16/600 Superdex 200 pg column (GE Heathcare Life Sciences) using TBS as the running buffer.

#### Immunisation

7–10-week-old female inbred BALB/c mice (Envigo, UK), or 7–12-week-old C57BL/6J or B6.129S6-*Nlrp3*^*tm1Bhk*^/J of any sex (bred in-house) were immunised i.m. with a total volume of 50 μL in the tibialis muscle under light isoflurane anesthesia. Each vaccine dose contained 1 μg R21 in endotoxin free low phosphate PBS and 25 μL of indicated adjuvants as described above.

#### Malaria challenge

Malaria challenge was performed as described previously.^17^ For all experiments 1,000 transgenic *P. berghei* sporozoites (described above) were injected intravenously (i.v.) in a total volume of 100 μL into the lateral tail vein of each mouse. From day 5 post challenge mice were monitored for infection by thin-film blood smear (fixed in methanol and stained in 10% Giemsa for 30 min). Mice were sacrificed when >1% parasitaemia was observed. If no parasites were detected on day 12 after challenge, mice were considered sterilely protected.

#### ELISA for serum isotypes against NANP (IgM, IgG, IgA)

ELISAs to detect antibodies against the central repeat region of CSP (NANP) were performed as previously described.^17^ Serum was obtained by collecting blood from the lateral tail vein in a microcuvette tube. Blood was allowed to clot at 4°C overnight before centrifugation at 13,000 rpm for 4 min and sera removed and stored at −20°C until use. For total IgG, IgM, and IgA ELISAs Nunc-Immuno Maxisorp 96 well plates were coated with 2 μg/mL NANP_6_ peptide (Mimotopes, NANPNANPNANPNANPNANPNANPC) in carbonate-bicarbonate coating buffer overnight at 4°C. Plates were washed with PBS-Tween (0.05% v/v) and blocked with 2% BSA in PBS-Tween for 1 h at RT. Sera were diluted appropriately between 1:100 and 1:30,000 (depending on number of immunisations and ± adjuvant) in 1% BSA in PBS-Tween and added to plate in duplicates. Plates were incubated for 2 h at room temperature and then washed as before. Fc-specific goat anti-mouse IgG conjugated to alkaline phosphatase (AP) (1 in 5,000, Sigma-Aldrich, A1418), or Goat Anti-Mouse IgM mu chain-AP (1 in 5,000, Abcam, ab98672), or Goat Anti-Mouse IgA-AP (1 in 2,500, Southern Biotech, 1040-04), was added for 1 h at room temperature. Following a final wash, plates were developed by adding *p*-nitrophenylphosphate at 1 mg/mL in diethanolamine buffer and OD was read at 405 nm. Total IgG concentrations against NANP in sera were calculated by interpolation against a standard curve of monoclonal 2A10 (MR4, MRA-183A). For IgM and IgA, a serum pool of previously R21-immunised mice was used to generate a standard curve. Sera from naive mice were used as negative control.

#### IgG subclass ELISA

To assess the quality of the antibody response against the central repeat region of CSP (NANP), IgG subclass ELISAs were performed as previously described.^56^ MaxiSorp plates (Nunc) were coated with 50 μL of 2 μg/mL NANP_6_ peptide (Mimotopes, NANPNANPNANPNANPNANPNANPC) in carbonate-bicarbonate coating buffer overnight at 4°C prior to washing in PBS-Tween (0.05% v/v) and blocking with 2% BSA in PBS-Tween for 1 h at RT. For detection of IgG subclasses, all serum samples were diluted to 80 ng/mL of total anti-NANP IgG in 1% BSA in PBS-Tween and added in duplicates. Plates were incubated for 2 h at 37°C and then washed as before. Afterward, AP-conjugated anti-mouse IgG subclass-specific secondary antibodies IgG1 (1 in 4,000, Southern Biotech), IgG2a (1 in 4,000, Southern Biotech), IgG2b (1 in 4000, Southern Biotech), IgG2c (1 in 4,000, Southern Biotech) or IgG3 (1 in 1,000, Abcam) were added and incubated for 1 h at 37°C. Following a final wash, plates were developed by adding *p*-nitrophenylphosphate at 1 mg/mL in diethanolamine buffer and OD was read at 405 nm. The results of the IgG subclass ELISAs are presented using optical density values.

#### T_H_1/T_H_2 index

To evaluate the T_H_1/T_H_2 index, serum titers of IgG1, IgG2a (BALB/c background), IgG2c (C57BL/6 background), and IgG3 were determined as described above. Afterward, the index was calculated as ([IgG2a or IgG2c+IgG3]/2)/(IgG1) as previously described.^44^ Increased T_H_1/T_H_2 index indicates T_H_1 skewed immune responses.

#### Avidity ELISA

Avidity of anti-NANP antibodies was assessed by chaotropic salt displacement ELISA as described previously.^43^ Serum samples whose anti-NANP total IgG titers had been determined previously were diluted to 80 ng/mL 50 μL of diluted sera were added to two columns of a Nunc-Immuno Maxisorp 96 well plate (Thermo Scientific) coated with 2 μg/mL NANP_6_ peptide (Mimotopes, NANPNANPNANPNANPNANPNANPC) in carbonate-bicarbonate coating buffer (Sigma-Aldrich). Plates were incubated for 2 h at room temperature, followed by washing and addition of increasing concentrations of NaSCN/PBS down the plate (0; 0,5; 1; 1,5; 2; 2,5; 3 and 3.5 M NaSCN). Plates were incubated for 15 min at room temperature, washed and Fc-specific goat anti-mouse IgG-AP (1 in 5,000, Sigma-Aldrich, A1418) added for 1 h at room temperature. Plates were developed by adding *p*-nitrophenylphosphate (Sigma-Aldrich) at 1 mg/ml in diethanolamine buffer (Sigma-Aldrich) and OD was read at 405 nm. Avidity is given as the IC_50_ of NaSCN (concentration of NaSCN at which the signal is half the intensity of the signal when no NaSCN was added).

#### Complement ELISA

Complement ELISAs were preformed to quantify the ability of anti-NANP antibodies in sera from vaccinated mice to bind C1q. MaxiSorp plates (Nunc) were coated with 50 μL of 2 μg/mL NANP_6_ peptide (Mimotopes, NANPNANPNANPNANPNANPNANPC) in carbonate-bicarbonate coating buffer overnight at 4°C prior to washing in PBS-Tween (0.05% v/v) and blocking with 2% BSA in PBS-Tween for 1 h at RT. Sera were diluted 1 in 100 in 1% BSA in PBS-Tween, added in duplicates to the top wells, diluted 2-fold down the plate, and incubated for 1.5 h at RT. After washing with PBS-Tween, plates were incubated with 50 μL of 1 μg/mL human C1q (Sigma-Aldrich, C1740) for 30 min at RT and washed again with PBS-Tween. Following incubation with goat anti-C1q-FITC (1 in 5000, Abcam, ab182940) for 1 h at RT, plates were washed with PBS-Tween and incubated with donkey anti-goat-IgG-AP (1 in 8,000, Abcam, ab6886). Following a final wash, plates were developed by adding *p*-nitrophenylphosphate at 1 mg/mL in diethanolamine buffer and OD was read at 405 nm. For analysis, summation of all dilutions absorbance was performed for each sample as described previously,^57^ to obtain the absorbance summation value (AS value of NANP C1q deposition). AS values of individual mice are plotted in the Figures.

#### ELISA for detection of cytokines in serum and muscle (SOI)

To detect innate cytokines in serum and muscle (SOI) samples, mice were immunised with R21 and indicated adjuvants as described above. For NLRP3 inhibition, 50 mg/kg MCC950 (CP-456773, Sigma-Aldrich, PZ0280-25MG) were injected i.p. 12 h and 1 h before immunisation. At time points indicated in Figure legend, mice were sacrificed, and tissues harvested. Blood samples were allowed to clot for 2 h before centrifugation at 13,000 rpm for 4 min. Sera were removed and stored at −20°C until use. Muscle samples were weighed and transferred into PBS supplemented with protease inhibitor (Roche, 11873580001). Afterward, samples were dissociated in gentleMACS M Tubes using the gentleMACS Octo Dissociator. Following transfer into a fresh tube and centrifugation at 10,000 rpm for 5 min, supernatant was removed and stored at −20°C until use. To detect cytokines and chemokines in these samples, the mouse macrophage/microglia multi-analyte flow assay kit (LEGENDplex, Biolegend) was used according to the manufacturer’s instructions. LSR II (BD) flow cytometer was used to assess fluorescence intensity of beads and LEGENDplex Data Analysis Software Suite was used to analyze the data.

#### Inhibition of Sporozoite Invasion Assay (ISI)

To assess the ability of sera to block sporozoite entry into hepatocytes *in vitro*, the ISI assay was performed as described previously.^58^ Mice were immunised with R21 and indicated adjuvants, and sera were obtained as described above. Huh7 (ATCC) hepatocyte cell line was propagated in DMEM (Dulbecco’s Modified Eagle’s Medium) supplemented with 10% heat inactivated FCS, 100 U/ml penicillin, 100 μg/mL streptomycin and 2 mM L-glutamine (all reagents obtained from Sigma-Aldrich). Cells were cultured at 37°C and 5% CO_2_ with the addition of TrypLE Express Enzyme (Life Technologies) to aid in detachment of cells from culture plates or flasks. 30,000 cells were seeded on 96 well-flat bottom plate 24 h prior to sporozoite addition. GFP expressing sporozoites (*PfCSP(r)PbCSP + GFP::Luc@Pbeef1a_230p*) were produced and harvested as described above. For addition of sporozoite to hepatocyte cell line, culture medium was removed and replaced with serum and sporozoite mixture (1 in 10 serum dilution, 15000 SPZ per well, final volume 100 μL), prior to centrifugation of the plates at 500 g for 5 min to enhance sporozoite entry into hepatocytes and incubation at 37°C.

Cells were harvested after overnight incubation and culture medium was removed from each well. 30 μL of trypsin (TrypLE Express Enzyme, Life Technologies) were added and incubated for 10–15 min, prior to resuspension in 1% BSA (10% Fetal Calf Serum) in PBS and transferred to a FACS tube for acquisition. 4′,6-diamidino-2-phenylindole dihydrochloride (DAPI, final concentration 1 μg/mL, Sigma-Aldrich) was added just prior to acquisition for the exclusion of dead cells. Samples were acquired with an LSRFortessa flow cytometer (BD Biosciences) using FACSDIVA software (BD Biosciences). *P*. *berghei* infected cells were identified by gating on viability and size, removing doublets and gating on GFP positive but APC (autofluorescence) negative cells in FlowJo software V10.8. Data are presented as % inhibition compared to the negative control where no serum was added during sporozoite infection of Huh7 cells.

#### Cell stimulation for *in vitro* inflammasome assays

Differentiated primary mouse BMDMs were plated at a density of 10^6^ cells/ml CMM media. Differentiated HMDMs were plated at a density of 0.7x10^6^ cells/ml CMM media. Cells were treated for 6 h with VFI adjuvants as indicated in Figure legends. For comparisons between WT BMDMs with inflammasome deficient BMDMs or HMDMs ± MCC950, cells were stimulated with VFI adjuvants at the highest concentration (1 in 20 dilution). Control cells were primed for 5 h (BMDMs) or 4 h (HMDMs) with 100 ng/mL *E.Coli* K12 ultrapure LPS (Invivogen, tlrl-peklps), and subsequently stimulated with 10 μM nigericin for 1 h (BMDMs) or 2 h (HMDMs). After cell stimulation, cell supernatants were collected and cells lysed for analysis as described below.

#### Readouts used to monitor inflammasome activity *in vitro* (cytokine, viability, caspase-1)

Secretion of IL-1β, IL-18 and TNF-α were monitored in cell-free supernatants using ELISA (Thermo Fisher, 88-7013-77, 88-7324-77 and BMS618-3 for mouse cytokines and 88-7261-77 and 88-7346-88 for human cytokines). Cellular viability was measured using cell culture supernatants and the Cytox96 nonradioactive cytotoxicity assay (Promega, G1780). Caspase-1 activity was measured in cell lysates and culture media by Western blot using anti-caspase-1 antibody (Casper-1, Adipogen, AG-20B-0042-C100 for mouse and D7F10, Cell Signaling 3866S for human) to detect p46 and p20 cleaved caspase-1. GAPDH (CST, 2118S) or Tubulin (Sigma-Aldrich, T5168) served as loading controls. Mouse and human NLRP3 were detected using Cryo-2 antibody (Caltag, AG-20B-0014-C100); mouse GSDMD (Abcam ab209845-100ul) and mouse GSDME (Abcam EPR19859).

#### Cell stimulation and readouts used to monitor CD4 T cell activity after vaccination

Mice of indicated genotypes were immunised with R21 and indicated adjuvants as described above. Spleens were collected 1 week after the second boost as indicated in Figure 4A, red blood cells lysed and splenocytes plated in 96 well plate at a density 10^6^ cells/ml. Plates were previously coated anti-CD28 (BioLegend 102112) and anti-CD49d (BioLegend 103709). A pool of 31 15-mer peptides overlapping by 11 amino acids spanning the *P. falciparum* CSP sequence present in R21 (Mimotopes, see key resource table for details) was added where indicated for 48 h. Cytokine secretion was measured using ELISA to detect IL-13 (Thermo Fisher, 88-7137-88) and IFN-γ (Thermo Fisher, 88-7314-77). Intracellular cytokine secretion in gated CD4^+^ T cells was measured using the following antibodies, anti-TNF-FITC (Biolegend 506304) and anti-IFN-γ-PE (BioLegend 505808). Samples were acquired with an LSRFortessa flow cytometer (BD Biosciences) using FACSDIVA software (BD Biosciences). To identify IFN-γ/TNF-α double-positive CD4^+^ T-cells, samples were pre-gated on size, removing doublets, CD3^+^, CD19^−^, CD8^−^ (all antibodies from Biolegend), using FlowJo software V10.8.

#### Statistical analysis

Prism Software V10 was used to perform statistical analysis. Number of replicates, applied statistical test, and pairwise comparisons with correction for multiple testing are indicated in Figure legends. In all experiments ∗p < 0.05, ∗∗p < 0.01, ∗∗∗p < 0.001, ∗∗∗∗p < 0.0001. Non-significant differences (n.s.) are generally not indicated.

## Data Availability

• Original western blot images have been deposited at Mendeley and are publicly available as of the date of publication. The DOI is listed in the key resources table. • Our work does not contain other large datasets of a standardized datatype (e.g., -seq, proteomics, crystallography). The source data summarised in the heatmap in the Figure 3C are shown in the Figure 3B. • Any additional information required to reanalyze the data reported in this paper is available from the lead contact upon request.

## Supplemental information

Document S1. Figures S1−S3

Document S2. Article plus supplemental information
