# Supplementary material for: Emulsion and liposome-based adjuvanted R21 vaccine formulations mediate protection against malaria through distinct immune mechanisms
Source: Cell Rep Med. 2023 Oct 31;4(11):101245. doi: 10.1016/j.xcrm.2023.101245 (PMC10694591; doi:10.1016/j.xcrm.2023.101245)
Supplement: Document S1. Figures S1−S3 [file mmc1.pdf]

Cell Reports Medicine, Volume *4*

Supplemental information

Emulsion and liposome-based adjuvanted R21 vaccine formulations mediate protection against malaria through distinct immune mechanisms

Sören Reinke, Eirini Pantazi, Gabrielle R. Chappell, Alexandra Sanchez-Martinez, Romain Guyon, Joannah R. Fergusson, Ahmed M. Salman, Anjum Aktar, Ekta Mukhopadhyay, Roland A. Ventura, Floriane Auderset, Patrice M. Dubois, Nicolas Collin, Adrian V.S. Hill, Jelena S. Bezbradica, and Anita Milicic

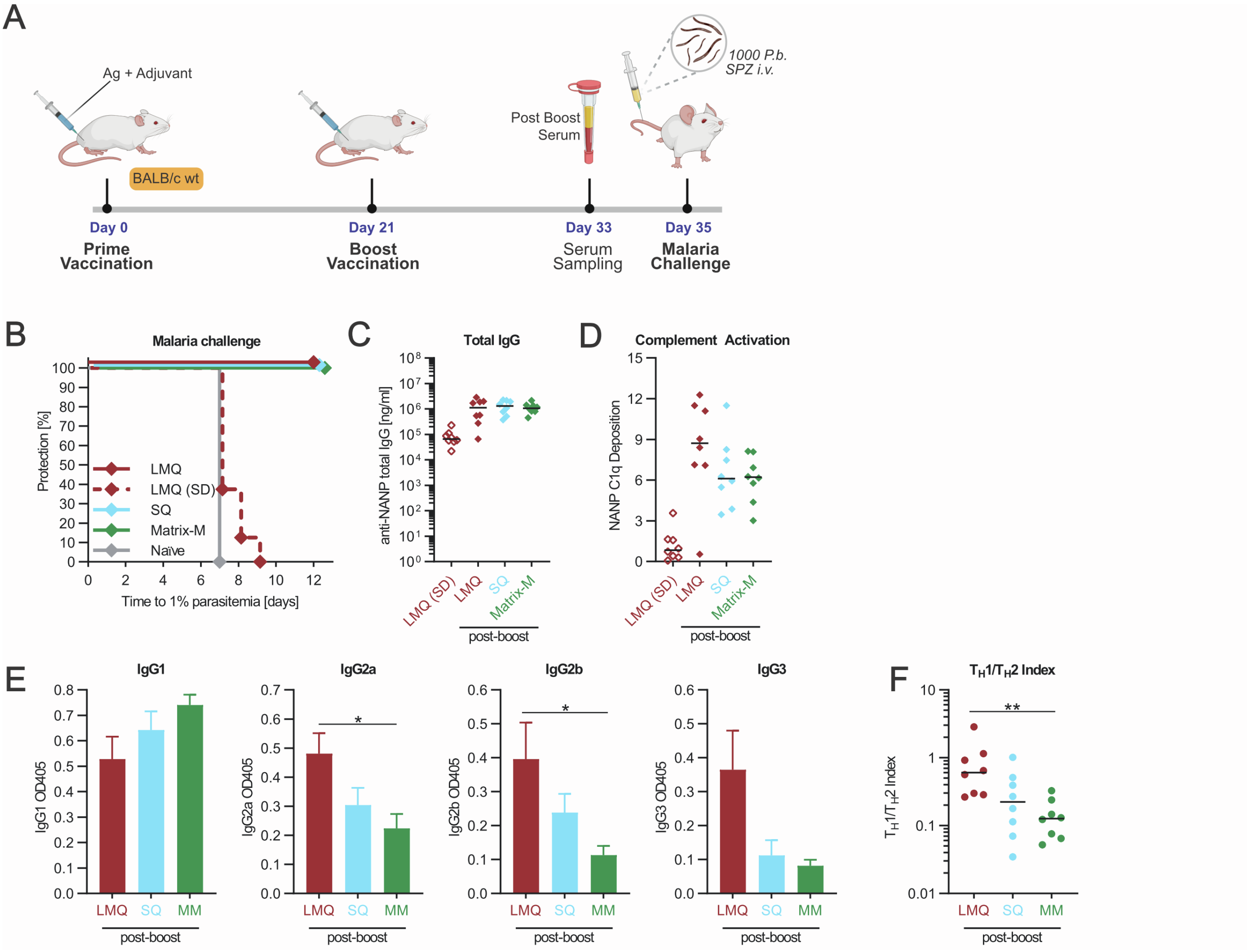

Figure S1 (Related to Figure 1): LMQ, SQ, and Matrix-M benchmark in malaria challenge.

A) Summary of the experimental protocol.

B) Kaplan-Meier curve of malaria challenge. BALB/c mice were vaccinated with R21 and indicated adjuvants before being challenged with malaria as described in Figure 1. SD: single dose of the priming vaccine only, for a comparison to the prime-boost regimen. (Data of one experiment; n=8).

C) Serum anti-NANP total IgG titres of mice challenged in (A) were measured as described in Figure 1. (Data of one experiment; median + replicates; n=8).

D) Complement activation of total serum anti-NANP antibodies, measured by C1q deposition assay. (Data of one experiment; median + replicates; n=8).

E) Anti-NANP IgG subclasses by proportional ELISA. (Mean + SEM; Kruskal-Wallis ANOVA with Dunn's multiple comparisons).

F) T_H1/T_H2 index of adjuvant-induced IgG subclasses calculated as ([IgG2a+IgG3]/2)/IgG1. Increased T_H1/T_H2 index indicates T_H1 skewed immune response. (Median + replicates; *$p < 0.05$, **$p < 0.01$, Kruskal-Wallis ANOVA with Dunn's multiple comparisons).

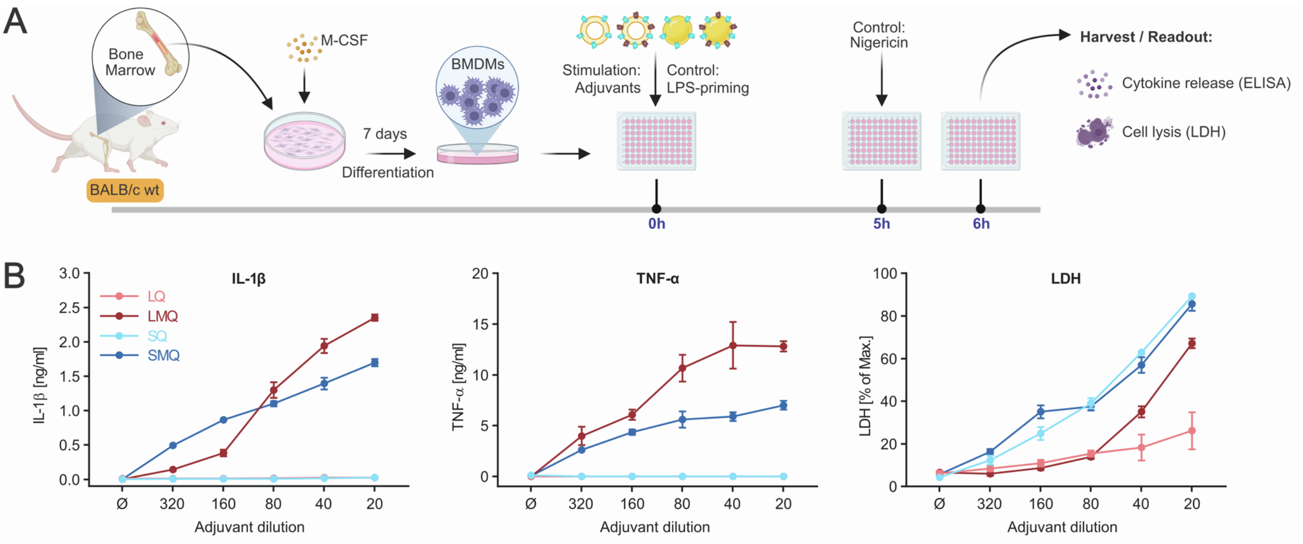

Figure S2 (Related to Figure 2): LMQ and SMQ induce an inflammasome signature in BALB/c WT BMDMs.

A) Summary of the experimental protocol. BMDMs were generated from the bone marrow of BALB/c WT mice. After adjuvant stimulation, cells were harvested and analysed for cytokine release and cell lysis.

B) IL-1β and TNF-α secretion in supernatants after stimulation of BALB/c BMDMs with indicated amount of adjuvants was determined by ELISA. LDH release was measured with a colorimetric assay and is depicted as a percentage of lysed positive control. (Data representative of three independent experiments; cells are stimulated in triplicates; mean ± SEM are shown).

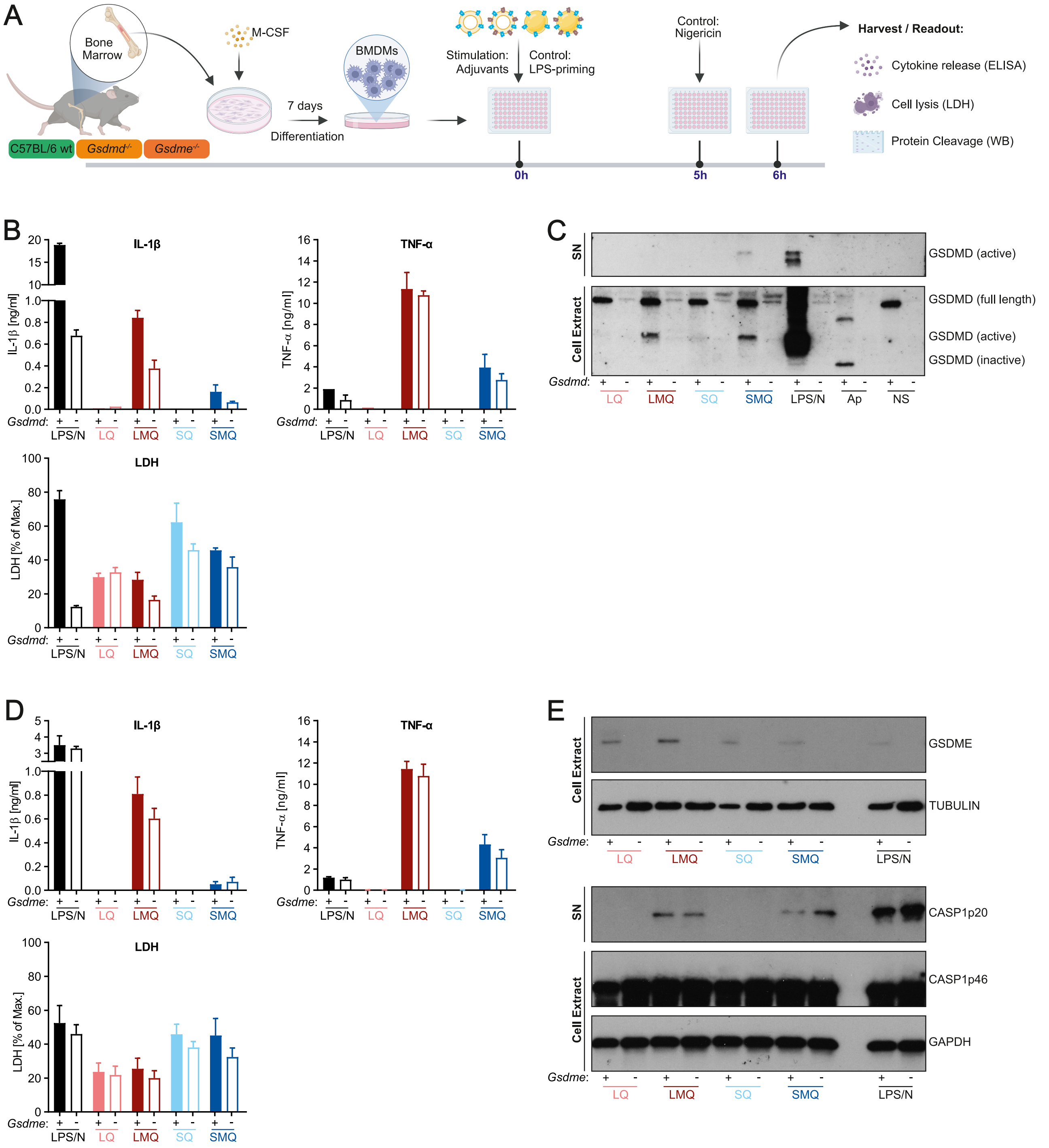

Figure S3 (Related to Figure 2): Gasdermins are partially involved in the MoA of LMQ and SMQ.

A) Summary of experimental protocol. BMDMs were generated from bone marrow of C57BL/6 WT, *Gsdmd*$^{-/-}$ and *Gsdme*$^{-/-}$ mice. Cells were harvested following adjuvant stimulation and analysed for cytokine release, cell lysis, and protein cleavage.

B) WT and *Gsdmd*$^{-/-}$ BMDMs were stimulated with adjuvants (1:20 dilution). IL-1β and TNF-α secretion in supernatants was measured by ELISA. LDH release was measured as in Figure S2. LPS/Nigericin (100 ng/ml LPS for 6 h with 5 µM nigericin for the last 1 h) stimulation was used as a positive control. (Data representative of three independent experiments; cells were stimulated in triplicates; mean ± SEM are shown).

C) Representative western blots for full length and cleavage fragments of GSDMD in cell lysates and supernatants from WT and *Gsdmd*$^{-/-}$ BMDMs stimulated with adjuvants (1:20 dilution) or LPS/Nigericin). Ap: apoptosis inducing drugs ABT-737 and S63845 (500 nM) which generate the inactive fragment of GSDMD.

D) WT and *Gsdme*$^{-/-}$ BMDMs were stimulated with adjuvants (1:20 dilution). Cytokines and LDH were measured as described in (B). LPS/Nigericin stimulation was used as positive control. (Pooled data from four independent experiments. Mean ± SEM n=4).

E) Representative western blots for GSDME, Tubulin, Caspase-1, and GAPDH in cell lysates and supernatants from WT and *Gsdme*$^{-/-}$ BMDMs stimulated with adjuvants (1:20 dilution) or LPS/Nigericin. (Data from one representative experiment of three).
